# Biofeedback-Based Proprioceptive Training to Improve Functional Prerequisites of Dragon Boating in Breast Cancer Survivors

**DOI:** 10.3390/ejihpe14050089

**Published:** 2024-05-11

**Authors:** Giuditta Carretti, Angela Dabraio, Mirko Manetti, Mirca Marini

**Affiliations:** Department of Experimental and Clinical Medicine, Section of Anatomy and Histology, University of Florence, 50134 Florence, Italy; giuditta.carretti@unifi.it (G.C.); angela.dabraio@edu.unifi.it (A.D.); mirko.manetti@unifi.it (M.M.)

**Keywords:** breast cancer-related sequelae, dragon boat, adapted physical activity, sensorimotor control, trunk stability, upper body proprioception, biofeedback training, sensorized proprioceptive board, kinesiologist, physical performance

## Abstract

Breast cancer (BC)-related sequelae drastically impact the psychophysical functioning and quality of life of affected women. Adapted physical activity (APA) has proved to effectively counteract these impairments in a non-medicalized framework. In particular, dragon boats are able to promote body functionality, social interaction, and quality of life in BC survivors, but the literature on specific motor gestures is scarce and practice is still based more on a re-educative perspective than a performative one. In this context, the present longitudinal study investigated the benefits of an adapted biofeedback-based sensorimotor training intervention on upper body functionality in a team of dragon ladies. The 8-week intervention was conceived as integrated dry workout sessions led by an APA kinesiologist and applied a novel sensorized proprioceptive device, such as a Libra board. Post-protocol evaluation revealed a significant improvement in bilateral upper limb mobility, core endurance, and trunk stability along with a distress decrease and quality of life enhancement through validated assessment tools. Our findings suggest that integrating biofeedback-based workout sessions can effectively promote upper body functionality in BC survivors practicing dragon boating. Furthermore, our innovative approach could help spread methodological hints able to boost exercise adherence in this target population, thus counteracting cancer recurrence while promoting overall well-being.

## 1. Introduction

Breast cancer (BC) is the most common neoplasm worldwide and the most frequently occurring in the global female population [1,2,3]. Despite the high incidence rate, thanks to medical and scientific advances, women with BC are increasingly surviving the disease, but almost 90% of them experience long-term sequelae following surgical and therapeutic treatments [4,5]. Specifically, symptoms may involve postural alterations, chronic pain, fatigue, sleep disorders, cognitive dysfunctions, and upper limb lymphedema along with a decline in strength, mobility, and aerobic capacity [6,7,8]. Despite the proven effectiveness of chemotherapy in cancer care and the steady progress in attenuating side effects, such medical therapy still implies several toxicity-related complicacies. Among them, musculoskeletal disorders and bone density loss are the most common affecting the postural and balance control of women undergoing BC treatment [9,10,11]. Moreover, in the case of secondary upper limb lymphedema onset, these individuals experience even worse sensorimotor deficits that further discourage any physical engagement, hence progressively limiting autonomy and functionality [12,13,14]. Regarding psychological and emotional well-being, BC survivors frequently experience depression-, anxiety-, body image-, and sexuality-related issues [15,16,17]. Oncological diagnosis and treatment oftentimes challenge the identity, self-esteem, body awareness, and relationships of the affected women, resulting in feelings of distress, uncertainty, and social isolation [18,19,20]. Given the significance of body image for female psychophysical and socioemotional health, such a multidimensional construct is frequently traceable as a main focus of the literature addressing this target population [21,22,23]. Specifically, self-appearance concerns are worth particular and sensitive attention and management for BC survivors due to their negative impacts on social life participation [24,25]. 

It is well known that human interaction is crucial for individuals dealing with cancer since it represents an important source of emotional support/comparison, disease-related stigma mitigation, self-acceptance, and motivation to overcome difficulties [26,27,28]. Many studies have proved the adverse impact of the aforementioned sequelae on overall health and self-efficacy in daily life/recreative activities; BC survivors hence deal with a drastic decrease in quality of life [29,30]. 

In order to counteract and prevent permanent psychophysical dysfunctions, a holistic approach and a tailored global management are needed and recommended for this target population [12,31,32]. Great variation is detected amongst oncological subjects due to individual factors such as age, gender, cancer subtype, disease stage, and pre-diagnosis health status and functional capacity. Despite this, the American College of Sports Medicine (ACSM) acknowledged, over the years, that supervised adapted training, regularly performed, can safely and effectively counteract cancer-related sequelae, improve psychophysical functioning, and prevent pathological recurrence [33,34]. Recently, the specific ACSM guidelines for exercise tolerance, testing, prescription, and delivery in cancer survivors have been updated according to the research findings and advances in this field [35,36]. These evidence-based recommendations highlighted the benefits of precise doses of aerobic or combined aerobic-resistance training on several psychophysical parameters and the overall health-related quality of life in individuals with cancer [37,38]. 

Particularly referring to BC survivors, it has been demonstrated that adapted physical activity (APA) protocols, conceived and led by a graduate specialist, should include progressive exercises focused on body schema re-education, proprioceptive postural awareness, balance, upper body sensorimotor control, and core stability/endurance [39,40,41]. Since sports participation naturally promotes body–mind involvement and social interaction, thus boosting exercise adherence over time in a non-medicalized context [42,43], some disciplines have been investigated and their technical fundamentals turned out to be particularly suitable to the complex needs of women with stabilized BC outcomes [44]. 

Among them, the literature has repeatedly highlighted the benefits of dragon boating on the overall health and quality of life in this target population [45,46]. The origins of such rowing activity are linked to an ancient Chinese ritual held to avert misfortune, and the name is inspired by the dragon heads and tails decorating the traditional paddled watercrafts. Nowadays, dragon boating is a worldwide practiced team-based discipline involving a vigorous cyclic motor gesture performed by the paddlers following a drum rhythm [47,48]. It has been shown that rowing is a complete water sport able to effectively improve the quality of life and psychophysical functionality of practitioners while promoting social interactions in a natural environmental frame [49,50,51]. Given the negative impact of cancer on all these parameters and the widely reported benefits of a regular physical activity engagement in oncological subjects, a version of this discipline exclusively addressing women with BC, therefore called “dragon ladies”, has been conceived in Canada in the early nineties [45,52]. This female sport discipline maintains the technical and performative features of the traditional one while simultaneously focusing on psychophysical and socioemotional post-disease re-education through a collective and supportive leisure context [53,54,55,56]. Currently, numerous teams regularly racing in the official competitions organized and acknowledged by the International Dragon Boat Federation are globally traceable [57]. 

Despite this worldwide interest in this sport and the increased performance level required of practitioners, the literature has not yet investigated the anatomofunctional prerequisites underlying sport-specific motor gestures. An athletic performance-oriented approach could speed up the return to normality, prevent injuries, and simultaneously boost the physical activity adherence and self-esteem of BC survivors [58,59]. Collectively paddling following an external rhythm while managing balance, strength, and coordination within an unstable water context requires finely orchestrated motor skills which need to be trained through a multimodal approach comprising total-body and segmental exercises [60,61]. Given the cyclic, coordinative, and strenuous nature of the rowing gesture peculiarly engaging upper body stability and upper limb mobility and strength, athletes should optimize its kinematics to increase paddle stroke efficacy and prevent traumatic/overuse injuries mostly due to the onset of fatigue [62,63]. Paddling technique effectiveness is strongly related to upper body sensorimotor control and, therefore, core stability/endurance, trunk–upper limb coordination and proprioceptive balance management over unstable surfaces should become crucial workout focuses [64,65]. Particularly for dragon ladies, athletic prerequisite training and evaluation should make use of not only traditional tools but also innovative ones purposely designed to optimize and foster body awareness without generating psychophysical overloads. The advent of new technologies allows us to investigate and quantify postural control efficiency according to the involved functional systems and recalling the multisensory nature of reality [66,67]. Specifically, these innovative tools consist of sensorized proprioceptive boards equipped with a biofeedback-based digital interface purposely designed to analyze the quality of static and dynamic postural control and objectively assess the global and segmental functional stability [68]. Such integrated biofeedback mechanism is able to provide continuous real-time information related to specific functional parameters by transducing them into visual, auditory, or somatosensory signals. Therefore, the subject can visualize them on the monitor or acoustically infer the micromovements of the whole body or its segments, progressively refining their perception, awareness, and control. Bringing normally undetectable input to a conscious and multisensory level can effectively promote anticipatory and reactive sensorimotor control, globally involving the subject and fastening motor learning processes [69]. It has been demonstrated that motor efficiency is strongly linked to the most archaic component of the proprioceptive system that can be productively stimulated and re-educated exclusively through a high-frequency proprioceptive input flow [70]. When variance is detected by the traditional tilting boards, the sensorized ones apply high-frequency signals able to train the subject to properly select and interpret them, thus progressively generating rapid and relevant sensorimotor responses [71,72]. Such a methodological approach also allows us to objectively evaluate the individual motor skills and consequently tailor each exercise aim/load to them [73,74]. Officially introducing dry workout sessions focusing on anatomofunctional prerequisites in the regular dragon boat training schedule grants safe and effective training for this target population, simultaneously promoting individual self-efficacy and team membership [56,75].

On this basis, the present longitudinal study investigated, for the first time, the possible benefits of an adapted and integrated biofeedback-based sensorimotor training intervention on upper body functionality in a Tuscan team of dragon ladies. Our intervention, peculiarly tailored to the BC survivor needs and dragon boat performance model, was designed and led by an APA graduate specialist legally known as an APA kinesiologist. Given the demonstrated biofeedback effectiveness on motor learning and control [76,77], we are confident that this study can hopefully provide novel methodological/evaluative hints specifically addressing dragon boat performance, thus further enriching knowledge in this field and shifting the perspective from a mere re-educative aim to a more sports performative one. 

## 2. Materials and Methods

### 2.1. Participants

A Tuscan team of dragon ladies received the proposal to take part in the present study directly promoted by the APA graduate specialist officially acknowledged within the team staff since 2018. The investigated dragon boat team is officially registered to Astro Onlus, a local association providing social support and re-education for oncological subjects through multidisciplinary activities in a non-medicalized context involving different professional figures, such as psychologists, music therapists, and kinesiologists. Hence, dragon boat is one of the available options of the aforementioned wide offer. Of note, a sport-specific 60 min dry workout session per week was habitually led by the APA specialist even before this biofeedback-based tailored intervention. Given the post-pandemic frame in which the sensorimotor protocol was carried out and the consequent not fully resumed workout schedule of the team, only 12 dragon ladies out of the 22 regularly registered to the crew before COVID-19 emergency provided their signed informed consent and voluntarily adhered to the training intervention and related assessments. Concerning any possible physical risk, all the subjects were in possession of a valid sport medical certificate issued by a sports doctor as is mandatory to take part in the dragon boat competitive practice. The present protocol was conceived as an integrative 90 min dry workout session per week not merely aimed at sport-specific athletic performance but also recalling the pre-COVID-19 training schedule organization of the team (i.e., one 60 min dry workout session focused on a sport-specific gesture and two 60 min dragon boat water training sessions per week). The study sample continued performing the two aforementioned water training sessions per week during the whole sensorimotor protocol investigation, regularly attending the integrative dry workout scheduled by the APA kinesiologist. As commonly provided for Italian sport associations, in the act of renewing the annual membership to the team, each athlete provided informed consent and agreed to participate in the training and testing activities promoted by the technical staff during the whole sports season. Since the present protocol was designed, proposed, supervised, and carried out by the official APA kinesiologist in a non-medicalized context after receiving approval from the team management and the investigated sample consisted of competitive dragon ladies regularly trained and evaluated by the abovementioned specialist during the sports season, no formal approval by an ethics committee was applicable. All study procedures were conducted following the rules of the Declaration of Helsinki of 1975 [78], revised in 2013. In agreement with the informed consent provided by all participants, the data were treated, processed, and stored in a completely anonymous form for the purposes of this study. In detail, the study sample comprised 12 women (mean age: 59.9 ± 8.36 years) with stabilized BC outcomes (i.e., women who concluded the medical and rehabilitative phases of the disease management because of the absence of known or suspected sequelae, such as cancer recurrence, lymphangitis, and moderate/severe heart failure, and were thus referred by their oncologist to an APA kinesiologist for a psychophysical re-education protocol in a non-medicalized context) officially signed up for the Tuscan dragon boat team who routinely practice this sport discipline at a competitive level. 

### 2.2. Participant Evaluations

All dragon ladies gave their consent to undergo qualitative and quantitative evaluations at baseline and after ending the adapted sensorimotor training intervention. Psychosocial and anatomofunctional parameters, which are implicated both in daily life self-efficacy and sport performance, were assessed through traditional and innovative validated tools. Specifically detailing the qualitative evaluation, all the women compiled an anonymous self-administered survey purposely designed to collect sociodemographic data concerning age, educational degree, and employment status, as well as anthropometric values such as weight and height. Current and pre-diagnosis sport practice expertise, referred either to dragon boating or different physical activities, was also inquired. In addition, a particularly BC-centered section aimed to collect data about the operated side, underwent surgery type, applied adjuvant therapies, and the eventual presence and degree of upper limb secondary lymphedema. The last section of our survey assessed the perceived pain intensity in specific body regions and the psychological distress level through validated visual tools such as the Numerical Rating Scale (NRS) [40,79] and distress thermometer [80], respectively. Both these tools consist of a 0–10 graphic scale with higher values corresponding to worse perceived pain and psychological distress levels, respectively [81]. Finally, the perceived quality of life was measured through the mental and physical indexes extrapolated from the 12-item Short-Form (SF-12) questionnaire, a qualitative assessment tool frequently used not only in healthy populations but also in the oncological field [82,83]. In particular, higher scores in the two aforementioned components are associated with a better quality of life [84]. The post-protocol survey also comprised questions inquiring the satisfaction level in the methodology and specific motor contents applied during our sensorimotor training intervention along with the graduate specialist’s degree of competence. 

Given the main objective of our study and the well-documented BC sequelae, the anatomofunctional quantitative evaluation particularly focused on upper body mobility, strength, and flexibility. In detail, the spine and upper limb active range of motion (AROM) were assessed using a digital postural goniometer. Aiming to investigate the motor patterns involved in the dragon boat-specific paddling gesture, these parameters were sequentially evaluated by asking the subject to perform a trunk flexion and rotation and an upper limb flexion, extension, abduction, and extrarotation. All the aforementioned movements have been performed and assessed bilaterally. 

With an aim to measure core strength/endurance and posterior muscle chain flexibility, sit-up and sit-and-reach tests were administered, respectively. Both assessment tests were conducted rigorously following the validated guidelines available in the literature [85,86]. Briefly, the sit-up test evaluates abdominal muscular endurance and strength by asking the subject to perform as many sit-ups as possible in 1 min. The instructions are as follows: start from a supine position on the floor, bend lower limbs with feet spread hip-width apart held down on the ground by the examiner, and keep straight parallel upper limbs forming a 45°-trunk angle, with palms facing the floor; on the examiner’s command, raise the upper body from the floor by activating abdominal muscles until knees are touched with both hand fingertips, then slowly return to the starting position. Since muscular endurance is generally expressed as the maximum amount of repetitions performed within a fixed period of time, the highest number of correctly executed sit-ups in 1 min is recorded [85]. The sit-and-reach test is a widely and easily applied tool to estimate hamstring and, in general, posterior muscle chain extensibility by measuring a fingertips-to-tangent feet distance in a straight leg seated position on the floor [86]. Such a lineal test is characterized by a simple procedure to administer and requires minimal training skills and equipment to perform. The assessment of BC survivors using the sit-and-reach test was carried out as detailed elsewhere [39,87]. 

Furthermore, latero-lateral trunk stability was investigated from a sensorimotor perspective using the sensorized proprioceptive board Libra, a validated digital device which we have already described in detail in a previous study [68]. Briefly, the disposal consists of a square unstable platform equipped with different tilting radius wedges and a USB connectable to a computer. The pre-installed software provides several training and testing settings and visual/acoustic feedback to help the investigated subject keeping the board parallel to the ground while following the pattern course set by the examiner. Opting for a complete feedback modality, the track is displayed on a computer screen, and different auditory signals are played when its boundaries are met or overcome. Within the present intervention, the evaluation with this innovative tool was conducted by setting up a seated position on the board and a high oscillation degree, thus recalling a specific position, dynamic postural control, and instability frame typically required during dragon boat technical gesture performance. In particular, Libra was set on its non-slip mat placed on a wood jump box, hence allowing the subject to safely seat over the board, keeping hands on hips, a 90° trunk–thigh angle, and both legs orthogonal to the floor and feet, hip-width, firmly on the ground. Depending on individual anthropometric parameters, jump box height was adjusted, thus granting each participant to respect and maintain the aforementioned body attitude. Concerning the test settings, the sensorized board was straight oriented (latero-lateral swing on frontal plane) and a 10 cm tilting radius wedges, a maximum difficulty level, a sinusoidal course pattern, and a 60 s duration were set up. The aim of the test was to keep the proprioceptive board in balance by adjusting body posture according to the visual and auditory feedback provided by the software. At the end of the test, the performance index was recorded into the database and then compared to the validated values supplied by the manufacturer. Since biofeedback-based technology implies a multisensory involvement not often naturally mastered, the graduate specialist scheduled and supervised a trial test aimed to show and explain the visual and acoustic peculiarities of Libra digital interface to each participant. 

### 2.3. Adapted Sensorimotor Training Intervention

The adapted protocol was conceived as an 8-week integrative workout aimed to improve upper body sensorimotor control through a biofeedback-based training of paddling anatomofunctional prerequisites in a Tuscan team of dragon ladies. Each 90 min dry session was scheduled once a week from March to May 2022 and supervised/led by an APA kinesiologist. In order to easily tailor exercise load to the individual needs/skills of each participant while simultaneously taking advantage of a group workout, a circuit training methodology was applied. In fact, such a methodological approach is based on a workout time scheduled for each circuit station instead of a preset number of repetitions, thus allowing subjects to collectively perform exercises while simultaneously respecting their subjective physical fitness level and adjusting the execution pace accordingly.

Every training session comprised a total body warm-up performed with music, hence enhancing rhythmic perception and workout enjoyment, followed by mobility exercises using small fitness tools such as elastic bands and sticks. The central part of the session consisted of 6-station circuit training, with two women simultaneously performing the same exercise for 2 min on each station, for a total of 2 complete circuit rounds. Given the biofeedback-based sensorimotor focus of our protocol, one station always involved exercise on Libra. Since the sensorized device only allows individual utilization on this station, one participant performed the exercise on it, while the other one used a traditional tool such as proprioceptive board, skimmy, or foam pad. During the second round of the circuit, once on the Libra station, women had to trade exercises from the previous round, thus allowing each subject to use the digital device. Finally, the last part of each workout session was focused on dynamic stretching, body awareness, and breathing exercises. 

**Figure 1:**
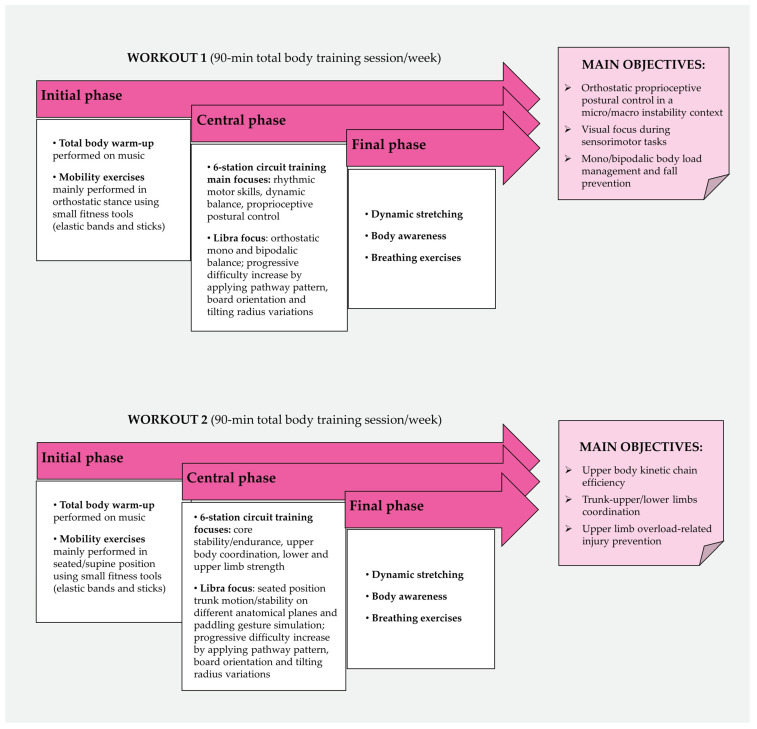
Adapted sensorimotor training organization.

Considering the main objective of our study and the complex multidimensional needs of BC survivors, the adapted protocol has been designed as a total-body dry workout integrating the two 60 min water training sessions per week regularly performed by the dragon boat team. Applying a high-frequency proprioceptive training based on biofeedback allowed us to safely quicken sensorimotor control acquisition, hence preventing any psychophysical overload and upper body injuries in this sensitive target of individuals [88,89]. To improve, in a dry context, the main motor patterns underlying dragon boat technical gesture and recall the dynamic balance skills required of practitioners, specific paddling simulation exercises on Libra were performed during the last sessions of the protocol. Moreover, aiming to boost trunk stability, high tilting and difficulty levels have been set up, thus promoting upper body sensorimotor control through small amplitude sway, pelvis stabilization, and core recruitment [90,91]. The training load has been progressively increased by varying body position during exercises, using light dumbbells, wrist/ankle weights or elastic bands, raising instability level, and requiring complex coordinative tasks. Two distinct circuit training schedules have been designed and alternately administered during the 8-week intervention, thus avoiding overuse syndromes affecting specific body regions. Particularly, one of the schedules was mostly focused on rhythmic motor skills, dynamic balance, and proprioceptive postural control improvement, whereas the other one aimed to increase core stability/endurance, upper body coordination, as well as lower and upper limb strength. Figure 1 graphically illustrates the aforementioned workout organization. Furthermore, in order to ease and promote motor contents reproducibility, 2 circuit training schedules are detailed and provided as online Supplementary Materials, with Supplementary Figures S1 and S2 specifically showing the digital interface and exercise settings of the sensorized Libra board.

### 2.4. Statistical Analysis

All data are represented as the mean ± standard error of the mean (SEM), mean ± standard deviation (SD), or number/percentage of subjects. The paired Student’s *t*-test was used to compare the baseline vs. post-adapted sensorimotor training (AST) intervention scores after verifying the normality of data with a Kolmogorov–Smirnov test and further confirmation with a Shapiro–Wilk test. Values of *p* < 0.05 were considered statistically significant. Statistical analyses were performed using the SPSS version 28.0 (Statistical Package for the Social Sciences, Chicago, IL, USA).

## 3. Results

Twelve BC female survivors practicing dragon boat (mean ± SD age: 59.9 ± 8.36 years) took part in this study. 

Focusing on the clinical baseline characteristics of BC survivors, the majority of the women had undergone a mastectomy with breast reconstruction (50%), followed by breast-conserving surgery (33.3%) and modified radical mastectomy (8.3%) (Table 1). The adjuvant treatment strategy included endocrine therapy (58.3%), radiotherapy (58.3%), and/or chemotherapy (41.7%) (Table 1). The numbers of participants with dominant and nondominant affected sides were closely represented; only 16.6% of subjects were bilaterally operated. Different stages of upper limb lymphedema were detected in 25% of women, while 75% did not present this BC-related sequela (Table 1).

Regarding educational level, 33.3% of the women had attained a university education, 43.7% had a high school degree, and 23% had a middle school degree. For employment status, most of the women (33.3%) were teachers, employees (25%), or retirees (25%), whereas 8.3% were freelancers and 8.3% health professionals. Concerning current physical/sport activities regular practice in addition to dragon boat, 50% of BC survivors participated in physical activities: 25% aquagym, 16.7% pilates/yoga, and 8.3% fitness. 

Table 2 shows the questionnaire qualitative data at baseline and post-AST intervention. In particular, a trend toward a decreasing pain perception in the different body region was observed. Of note, a significant improvement in cervical spine pain was recorded. However, a significant increase in pain, referred to the breast area, was observed post-AST, as well as shoulder and upper limb pain in the paddling side though not reaching the statistical significance (Table 2). A trend toward improvement in the distress thermometer was also found post-AST (Table 2). Finally, the higher than baseline SF-12 mean scores for both the physical and mental components achieved post-AST intervention highlighted a trend toward a better quality of life (Table 2).

The results of anatomofunctional assessments are reported in Table 3. In detail, bilateral upper limb AROM values showed a statistically significant improvement post-intervention in flexion, abduction, and extrarotation movements. In the extension, a trend toward a symmetric improvement was also observed (Table 3). Furthermore, the right/left lateral flexion showed a mild improvement, though not statistically significant, following the AST intervention. Conversely, post-AST intervention, a reduction in rotation with respect to baseline was observed. In particular, the right rotation was significantly decreased (Table 3). Notably, the trunk stability index, assessed by the sensorized Libra board, resulted in significantly improved values (Table 3). In addition, a significant improvement in core strength, evaluated with the sit-up test, was observed (Table 3). Finally, the posterior muscle chain flexibility, measured by the sit-and-reach test, was also significantly increased following the specific AST program (Table 3). 

## 4. Discussion

To our knowledge, the current study is the first to provide direct evidence that a well-planned, structured, and adapted biofeedback-based sensorimotor training can improve the functional prerequisites of dragon boating in BC survivors. In particular, our findings demonstrated a significant improvement in bilateral upper limb mobility, core endurance, and trunk stability, along with a decrease in distress and a better quality of life.

Despite the steadily increasing survival rate, BC occurrence is a weighty event deeply compromising the psychophysical functionality of the affected subjects, especially due to treatment-related sequelae [4,92]. Since the resultant symptoms can emotionally and physically burden the daily life of BC survivors, with a drastic decrease in their perceived quality of life [30,93], the promotion of healthy habits that involve regular physical activity becomes essential to minimize and counteract such a complex clinical status [94,95,96,97]. Quality of life is inextricably linked to physical function, and it is well known that both are at high risk of decline following oncological therapies [98]. Specifically, a recent study reported that almost 60% of women deal with short- and long-term upper body issues post-BC surgery and adjuvant therapies [99]. This anatomofunctional morbidity is typically characterized by sensory and/or motor dysfunctions such as pain, muscle stiffness, weakness, limited joint mobility, movement pattern alterations, poor neuromuscular coordination, soft tissue fibrosis, and upper limb swelling and impairment [100,101]. All these symptoms deeply alter upper body functionality, thus adversely influencing daily life activities, social interaction, self-esteem, and autonomy [102,103,104]. From a psychological perspective, structured physical activity represents a consolidated and effective tool for anxiety/depression management and prevention, as well as mood, self-efficacy perception, and socialization promotion [105,106,107]. 

Even though leisure and sport activities specifically addressing this vulnerable target population are more oriented to overall psychophysical well-being recovery than to performance [108], in the case of stabilized outcomes, sport-specific athletic training and mixed strength–resistance exercise benefits have been demonstrated over time [33,109,110,111]. Supervised progressive exercise plays a crucial role in correcting/mitigating treatment-related postural alterations and muscle recruitment imbalance, hence helping to prevent injuries and upper body morbidity exacerbation [40,81]. Particularly, proprioceptive exercises targeted to this anatomofunctional region can effectively re-educate sensorimotor patterns and postural awareness by enhancing muscle flexibility, joint stability, and neuromuscular coordination [91,112,113]. 

The fundamental motor gestures and performance model of dragon boat particularly involve global and segmental sensorimotor control in a challenging framework, thus requiring practitioners to master the psychological and physical skills that need to be trained both individually and collectively [49,114,115]. Moreover, given the unstable and noisy outdoor environment in which this sport discipline is usually performed, specific athletic training sessions should regularly include proprioceptive exercises based on multimodal feedback to recall the real performative framework and boost multisensory integration and focus [76,116,117]. 

According to the aforementioned evidence and applying an innovative perspective simultaneously oriented to daily life and sport performance improvement, the main focus of our adapted athletic training intervention was to promote the upper body functionality of BC survivors practicing dragon boating through a proprioceptive multimodal approach. Since trunk postural control and segmental coordination cannot be increased without reinforcing global body awareness, anatomofunctional prerequisites, and conditional skills [17,71,118,119], a total body circuit training methodology has been applied. The use of a biofeedback-based tool, purposely designed for proprioceptive quantitative evaluation/improvement [68], and a tailored exercise load progression allowed us to reach the main goal of our intervention taking into account the individual skills and fitness level of each participant. Because of this methodological approach and the expertise of the graduate specialist in APA for oncological subjects, crucial prerequisites such as upper limb AROM, core endurance/strength, and posterior muscle chain flexibility have been improved. In detail, bilateral shoulder mobility in flexion, abduction, and extrarotation movements and sit-up and sit-and-reach test scores showed a statistically significant improvement post-AST protocol. Despite this bilateral upper limb mobility gain, a perceived pain enhancement, assessed using NRS, was detected in the paddling side shoulder and breast area during post-AST evaluation. Given the progressive and tailored exercise load applied to the whole training intervention, this qualitative outcome might be due to the post-COVID-19 framework in which the present investigation was carried out [120]. In fact, the literature has widely demonstrated that the recent pandemic and the consequent anti-contagion restrictions led to a drastic change in lifestyle behaviors, thus causing a massive physical fitness decline in all ages of the global population [121,122]. Several studies highlighted that these confinement-related detraining issues have been even worse and hard to counteract when referred to a vulnerable target such as oncological individuals [81,123]. Though gradually resumed, dragon boat training might simply have evidenced this phenomenon particularly referred to the anatomical regions most involved in paddling-specific motor gestures. Conversely, a post-intervention perceived pain decrease in the thoraco-lumbar region was detected, hence highlighting the benefits of proprioceptive and mobility exercises on postural control, body load management, and neuromuscular coordination [124,125,126,127]. Many daily living and recreative/sport activities require a strenuous exertion over a prolonged period of time; therefore, muscle endurance becomes a crucial requisite of physical performance and overall functionality [128,129]. In line with the current literature, core endurance plays a key role in controlling trunk motions over the pelvis during almost all daily-life and sport motor patterns, hence allowing an optimal force transfer along the kinetic chain while counteracting upper body overload and injuries usually due to the onset of fatigue [130,131,132,133]. A regular schedule of exercises focused on core strength/endurance, posterior muscle chain flexibility, and postural awareness might have played the speculated beneficial role [134,135]. Our data concerning trunk mobility, even though not statistically significant, may suggest an overall stability enhancement obtained through a mobility decrease in this body segment during rotation and a simultaneous increase during lateral flexion [136,137,138]. Specifically, core muscle recruitment, endurance, and strength, promoted using both traditional and innovative unstable tools while recalling paddling movement patterns, might have contributed to the abovementioned results [139,140,141]. Moreover, our intervention’s effectiveness on sensorimotor upper body control was further highlighted by the statistically significant improvement in latero-lateral trunk stability objectively assessed through the targeted test performed on the sensorized Libra board. Postural control, especially if sports performance framed, results from a complex selection and integration of vestibular, visual, proprioceptive, neuromuscular, and cognitive inputs [142,143]. Consequentially to a pathological condition and the subsequent medical treatments, all these information sources may be reduced or damaged, hence affecting self-efficacy in daily life and recreative/sport activities [14,144,145]. In such a context, sensorimotor augmentation training through a biofeedback-based device can be useful to reactivate and re-educate these systems, thereby counteracting their disease-induced weakness [76]. Even though the literature investigating such a topic is still scarce, some recent studies evidenced that providing real-time sensorimotor feedback of body displacement during balance training can effectively reduce body sway with a consequent promotion of global and segmental stability [117,146,147,148]. Therefore, the ability of detecting and, accordingly, self-correcting postural errors while performing motor tasks in an unstable and multimodal environmental frame should become a determinant training focus.

Given the BC-related sequelae and the sensorimotor prerequisites underlying dragon boat performance, our adapted intervention specifically focused, for the first time, on the multisensory biofeedback of trunk sway as an effective parameter for training segmental postural control and upper body efficiency [88,90]. Finally, regarding the psychophysical parameters investigated, a trend toward a decrease in distress along with a simultaneous mild increase in the perceived quality of life has been observed after participating in AST. In particular, both the physical and mental components of SF-12 questionnaire showed an improvement that, even though not statistically significant, confirmed the well-known adapted motor activity benefits on the overall health of BC survivors [12,149,150]. The considerable increase in the mental component becomes particularly relevant when considering the pandemic-related psychological sequelae particularly experienced by this sensitive target population [123] and the immediately post-event frame of the present study. Despite being limited to a single dragon boat team, the aforementioned results may deliver unprecedented insights into the psychophysical benefits of a biofeedback-based training tailored to the multidimensional needs of oncological individuals. 

## 5. Conclusions

In conclusion, our findings highlighted that integrating a regular training schedule with specific workout sessions aimed at segmental sensorimotor control improvement [151] using traditional and sensorized proprioceptive tools can effectively promote upper body functionality in BC survivors practicing dragon boating. Furthermore, the officially acknowledged introduction of an APA kinesiologist within the multidisciplinary teams addressing oncological subjects might provide effective and enjoyable sensorimotor re-educative approaches in a non-medicalized context [152,153]. Finally, by applying a biofeedback-based tool in a novel daily life and sports performance enhancement perspective, the present study could hopefully help spread innovative methodological ideas to apply and enrich in future research on larger scale.

## Figures and Tables

**Table 1:** Baseline characteristics of study participants.

Variables	Participants (*n* = 12)
Breast surgery, *n* (%)	
Quadrantectomy	4 (33.3)
Modified radical mastectomy	1 (8.3)
Radical mastectomy	1 (8.3)
Mastectomy with breast reconstruction	6 (50)
Controlateral recontruction	2 (16.6)
Lymph nodes removed	8 (66.7)
Operated side, *n* (%)	
Right	5 (41.7)
Left	5 (41.7)
Bilaterally	2 (16.6)
Adjuvant treatments, *n* (%)	
Chemotherapy	5 (41.7)
Radiotherapy	7 (58.3)
Endocrine treatment	7 (58.3)
Degree of lymphedema, *n* (%)	
None	9 (75)
Mild	2 (16.7)
Moderate	1 (8.3)

**Table 2:** Mean scores of the pain perception, distress thermometer, and quality of life questionnaires of dragon ladies (*n* = 12) at baseline and post-adapted sensorimotor training (AST) protocol.

Variables	Baseline Mean ± SD (SEM)	Post-AST Mean ± SD (SEM)	*p*-Value *
Pain perception (NRS)			
Shoulder	1.66 ± 2.14 (0.61)	2.33 ± 2.77 (0.80)	0.255
Upper limb	1.08 ± 1.97 (0.57)	1.16 ± 2.32 (0.67)	0.915
Chest	0.50 ± 1.24 (0.35)	1.25 ± 2.30 (0.66)	0.169
Breast area	1.33 ± 1.82 (0.52)	2.33 ± 3.02 (0.87)	0.020
Cervical	2.58 ± 2.60 (0.75)	1.41 ± 2.23 (0.64)	0.052
Dorsal	1.00 ± 1.75 (0.50)	1.16 ± 2.36 (0.68)	0.838
Lumbar	3.58 ± 2.74 (0.79)	3.33 ± 2.80 (0.81)	0.773
Distress thermometer	3.00 ± 2.98 (0.86)	2.33 ± 2.64 (0.76)	0.489
Quality of life (SF-12)			
Physical component	47.87 ± 8.01 (2.31)	48.26 ± 8.00 (2.31)	0.833
Mental component	42.91 ± 7.96 (2.15)	46.89 ± 7.96 (2.29)	0.067

Abbreviations: AST, adapted sensorimotor training; NRS, Numerical Rating Scale; SD, standard deviation of the mean; SEM, standard error of the mean. * Student’s *t*-test for paired data.

**Table 3:** Anatomofunctional assessment scores at baseline and post-adapted sensorimotor training (AST) protocol.

Variables	Baseline Mean ± SD (SEM)	Post-AST Mean ± SD (SEM)	*p*-Value *
AROM right upper limb, degrees			
Flexion	158.08 ± 19.74 (5.69)	161.66 ± 17.14 (4.95)	0.015
Abduction	147.41 ± 28.68 (8.28)	152.16 ± 27.37 (7.90)	0.003
Extrarotation	69.58 ± 13.22 (3.81)	72.00 ± 12.12 (3.49)	0.014
Extension	45. 66 ± 8.8 (2.55)	46.41 ± 7.90 (2.28)	0.202
AROM left upper limb, degrees			
Flexion	158.50 ± 18.15 (5.24)	160.66 ± 16.89 (4.87)	0.005
Abduction	151.08 ± 23.99 (6.92)	154.58 ± 23.23 (6.70)	0.001
Extrarotation	67.33 ± 13.68 (3.95)	69.25 ± 14.29 (4.12)	0.014
Extension	45.66 ± 7.41 (2.14)	46.41 ± 6.05 (1.74)	0.169
AROM trunk, degrees			
Right lateral inclination	52.75 ± 7.12 (2.05)	54.16 ± 7.00 (2.02)	0.157
Left lateral inclination	52.16 ± 7.99 (2.30)	53.00 ± 8.88 (2.56)	0.288
Right rotation	67.00 ± 9.66 (2.79)	60.83 ± 8.52 (2.46)	0.022
Left rotation	68.66 ± 10.64 (3.07)	67.50 ± 9.62 (2.77)	0.675
Sit-up, *n* repetition/min	27.58 ± 7.25 (2.09)	36.58 ± 15.24 (4.40)	0.024
Sit-and-reach, centimeters	1.00 ± 10.4 (3.00)	4.83 ± 8.5 (2.44)	0.001
Libra			
Trunk stability index	1.94 ± 1.28 (0.37)	1.36 ± 0.9 (0.26)	0.023

Abbreviations: AST, adapted sensorimotor training; AROM, active range of motion; SD, standard deviation of the mean; SEM, standard error of the mean. * Student’s *t*-test for paired data.

## Data Availability

All relevant data are included within this manuscript.

## Supplementary Materials

The following supporting information can be downloaded at: https://www.mdpi.com/article/10.3390/ejihpe14050089/s1, Examples of circuit workout schedules. Figure S1: Digital interface of the sensorized proprioceptive Libra board: orthostatic exercise settings; Figure S2: Digital interface of the sensorized proprioceptive Libra board: seated position exercise settings.
