# Supplementary material for: Biofeedback-Based Proprioceptive Training to Improve Functional Prerequisites of Dragon Boating in Breast Cancer Survivors
Source: Eur J Investig Health Psychol Educ. 2024 May 11;14(5):1351–68. doi: 10.3390/ejihpe14050089 (PMC11120340; doi:10.3390/ejihpe14050089)
Supplement: Supplementary file 1 [file ejihpe-14-00089-s001.zip › ejihpe-2963392-supplementary.pdf]

Supplementary Materials

CIRCUIT 1 WORKOUT SCHEDULE

- **Methodological approach:** total body circuit training
- **Specific focuses:** rhythmic motor skills, dynamic balance, and proprioceptive postural control
- **Number of stations:** 6 (2 subjects for each station)
- **Exercise time on each station:** 2 minutes (no recovery during station transitioning)
- **Total circuit rounds:** 2

Station 1: Low intensity simulated jump rope movement performed by visually and auditory following the pace of the exercise mate. Change jump technique (joined feet and alternate footstep) and pace provider role at each circuit round. Focus on feet to ground reactivity and pair rhythm synchronization.

Station 2: Orthostatic position, feet hip-width apart joined at the metatarsal level by a medium-resistance elastic loop band. Perform a thigh flexion until reaching a 90° thigh-pelvis angle; alternate lower limbs and contemporarily coordinate the contralateral upper limb as recalling the running motor gesture. Maintain for a few seconds the 90° thigh flexion position and focus on controlling/counteracting the instability caused by the elastic band resistance while foot descent to the ground. Elastic resistance level, execution speed and support surface instability degree could be progressively increased.

Station 3: Orthostatic position, feet hip-width apart, hold a stick with both hands and, keeping it parallel to the ground, perform a bilateral upper limb flexion until placing the stick overhead. Maintaining this position, shift the weight of one foot to the metatarsal (plantar flexion) and, simultaneously, that of the other on the heel (dorsal flexion) by performing a full roll. Combine such a motor combination with coordinated head rotations around the longitudinal axis of the body. Support surface instability degree as well as coordinative task difficulty could be progressively increased.

Station 4: Orthostatic monopodal position on a foampad, hands resting on hips. Perform a frontal plane abduction of the free lower limb while maintaining a slight dorsiflexion of the homolateral foot. Slowly come back to the starting position without placing the foot on the ground and, immediately afterwards, perform a 90° thigh-trunk flexion and touch the raised knee with the palm of the contralateral hand. Slowly come back to the starting position and repeat the entire motor sequence by changing the supporting limb. Execution speed, coordinative task difficulty could be progressively increased. In order to involve/improve the focus component, motor-cognitive dual tasks could be progressively added as well.

Station 5: Orthostatic position, spread lower limbs apart on the sagittal plane with the front foot placed on a skimmy, hold a stick with both hands and place it behind the head resting on trapezius. Perform a sagittal lunge while maintaining spine-posterior lower limb alignment perpendicular to the ground during the descent. Change the supporting limb on the skimmy at each circuit round. Instability degree could be progressively increased by placing the back foot on a foampad or adding upper body coordinative tasks.

Station 6: Bipodal orthostatic balance on Libra sensorized board 45° left oriented (combined swings in multiple planes), 24-cm tilting radius wedges, triangular wave pathway pattern with vertical scroll, difficulty degree 8/10. Keep the board in balance by following the software provided multisensory

biofeedback (Figure S1). Pathway pattern, tilting radius wedges, board orientation and difficulty degree could be progressively increased. The station mate was asked to perform the exercise on a traditional proprioceptive board while imaging to run the same pathway pattern set on Libra.

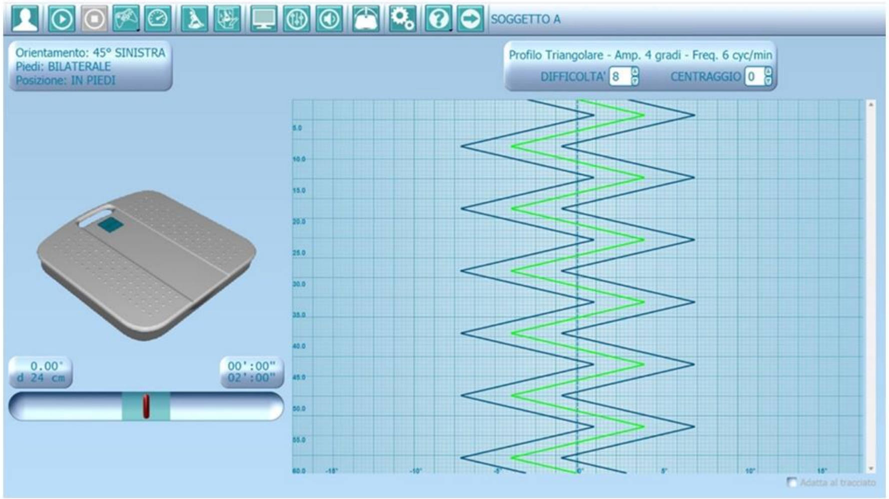

Supplementary Figure S1. Digital interface of Libra sensorized proprioceptive board: orthostatic exercise settings.

CIRCUIT 2 WORKOUT SCHEDULE

- **Methodological approach:** total body circuit training
- **Specific focuses:** core stability/endurance, upper body coordination, lower and upper limb strength
- **Number of stations:** 6 (2 subjects for each station)
- **Exercise time on each station:** 2 minutes (no recovery during station transitioning)
- **Total circuit rounds:** 2

Station 1: Bipodal orthostatic position on a foampad, feet hip-width apart placed over an elastic loop band handled on each body side keeping the upper limbs straight (grabbing hand palm facing the homolateral thigh). Perform alternating right-left trunk tilts on the frontal plane while maintaining head-torso postural alignment and controlling/counteracting the elastic resistance. This latter as well as the support surface instability degree could be progressively increased.

Station 2: Orthostatic position on a foampad with feet spread apart and slightly extra rotated, reach and hold an isometric semi-squat position. Arms adhered to body sides with forearms 90° flexed, hand palms facing each other and back joined by a medium-resistance elastic loop band. Maintaining this position, perform an extra rotation with both upper limbs while controlling/counteracting the elastic band resistance. This latter degree and upper limb coordinative task difficulty could be progressively increased. In order to involve/improve the focus component, motor-cognitive dual tasks and visuo-postural constraints could be progressively added as well.

Station 3: Seated position on a Swiss ball, feet hip-width apart placed on the floor and joined, at the metatarsal level, by a medium-resistance elastic loop band. Alternate right-left thigh flexion by raising foot from the ground and

simultaneously perform an ipsilateral-oriented trunk rotation around the longitudinal axis. During this rotational movement, keep upper limbs 90° abducted on the frontal plane, both forearm-arm full flexion (elbows kept at the shoulder level), and hand palms facing the floor. Control/counteract the elastic resistance during foot descent to the ground. Elastic resistance degree and upper body coordinative task difficulty could be progressively increased. Paddling motor gesture simulation could be performed as well.

<u>Station 4</u>: Supine decubitus position with lower limbs semi-flexed and feet placed on a skimmy, straight upper limbs lying on body sides, hand palms in contact with the floor. Raise the pelvis through a hip thrust movement while keeping the scapular area and upper limbs on the ground and the longitudinal axis of both legs perpendicular to the ground. Slowly come back to the starting position and, subsequentially, repeat the movement in monopodal support on the skimmy with contralateral limb extended, keeping knees parallel and not in contact with each other. Slowly come back to the starting position and repeat the whole motor sequence changing the supporting foot on the skimmy. Coordinated or crossed upper limb movements could be progressively added.

<u>Station 5</u>: Seated position on a skimmy placed on the floor with semi-flexed lower limbs and dorsiflexed feet hip-width (ground support by heels-floor contact). Place a medium-resistance elastic loop band under the feet soles and handle it at body sides (sledge riding position). Perform a 45° backward inclination of the trunk and, keeping the upper limbs straight and the loop band in traction, perform alternating trunk rotations around the longitudinal axis. Elastic resistance degree, support surface instability level and upper-lower body coordinative task difficulty could be progressively increased.

<u>Station 6</u>: Seated position on Libra sensorized board placed on a jump box, straight oriented (swings on the frontal plane),10-cm tilting radius wedges, sine wave pathway pattern with vertical scroll, difficulty degree 7/10. Perform the paddling motor gesture keeping Libra board in balance by following the software provided multisensory biofeedback (Figure S2). Pathway pattern, tilting radius wedges, board orientation and difficulty degree could be progressively increased. The station mate was asked to perform the exercise on a traditional proprioceptive board while imaging to run the same pathway pattern set on Libra.

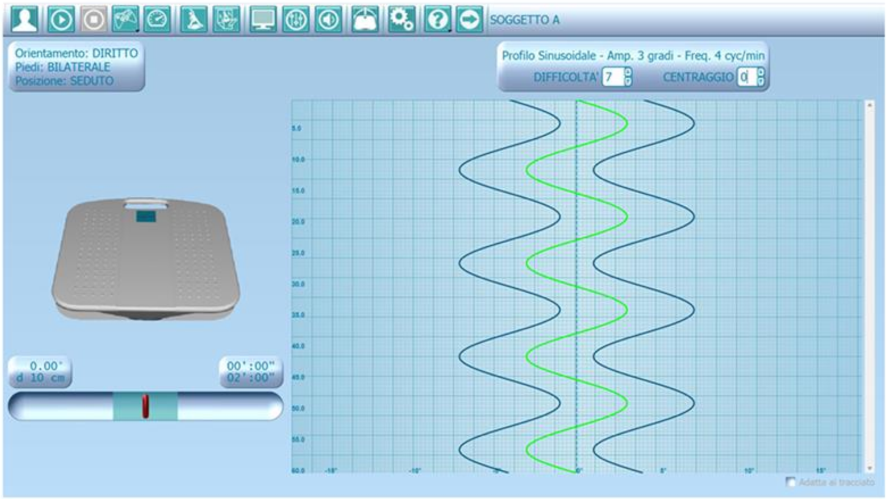

Supplementary Figure S2. Digital interface of Libra sensorized proprioceptive board: seated position exercise settings.
